# Comparative transcriptomic analysis of follicle-enclosed oocyte maturational and developmental competence acquisition in two non-mammalian vertebrates

**DOI:** 10.1186/1471-2164-11-18

**Published:** 2010-01-08

**Authors:** Maella Gohin, Julien Bobe, Franck Chesnel

**Affiliations:** 1 CNRS/IGDR (UMR 6061), IFR140 GFAS, Université de Rennes I, 2, Avenue du Pr. Léon Bernard, 35043 Rennes Cedex, France; 2 Institut National de la Recherche Agronomique, INRA SCRIBE, UR1037, IFR140 GFAS, Campus de Beaulieu, 35000 Rennes Cedex, France

## Abstract

**Background:**

In vertebrates, late oogenesis is a key period during which the oocyte acquires its ability to resume meiosis (*i.e*. maturational competence) and to develop, once fertilized, into a normal embryo (*i.e*. developmental competence). However, the molecular mechanisms involved in these key biological processes are far from being fully understood. In order to identify key mechanisms conserved among teleosts and amphibians, we performed a comparative analysis using ovarian tissue sampled at successive steps of the maturational competence acquisition process in the rainbow trout (*Oncorhynchus mykiss*) and in the clawed toad (*Xenopus laevis*). Our study aimed at identifying common differentially expressed genes during late oogenesis in both species. Using an existing transcriptomic analysis that had previously been carried out in rainbow trout, candidate genes were selected for subsequent quantitative PCR-based comparative analysis.

**Results:**

Among the 1200 differentially expressed clones in rainbow trout, twenty-six candidate genes were selected for further analysis by real-time PCR in both species during late oogenesis. Among these genes, eight had similar expression profiles in trout and *Xenopus*. Six genes were down-regulated during oocyte maturation (*cyp19a1, cyp17a1, tescalcin, tfr1, cmah, hsd11b3*) while two genes exhibited an opposite pattern (*apoc1, star*). In order to document possibly conserved molecular mechanisms, four genes (*star, cyp19a1, cyp17a1 *and *hsd11b3*) were further studied due to their known or suspected role in steroidogenesis after characterization of the orthology relationships between rainbow trout and *Xenopus *genes. *Apoc1 *was also selected for further analysis because of its reported function in cholesterol transport, which may modulate steroidogenesis by regulating cholesterol bioavailability in the steroidogenic cells.

**Conclusions:**

We have successfully identified orthologous genes exhibiting conserved expression profiles in the ovarian follicle during late oogenesis in both trout and *Xenopus*. While some identified genes were previously uncharacterized during *Xenopus *late oogenesis, the nature of these genes has pointed out molecular mechanisms possibly conserved in amphibians and teleosts. It should also be stressed that in addition to the already suspected importance of steroidogenesis in maturational competence acquisition, our approach has shed light on other regulatory pathways which may be involved in maturational and developmental competence acquisitions that will require further studies.

## Background

Late oogenesis is a key period in the complex process ultimately leading to the release of the female gamete from the ovary. During this period, the oocyte undergoes meiotic maturation (also known as oocyte maturation), a process leading to an oocyte that will remain arrested in metaphase II until fertilization. The ovulatory process that allows the release of the oocyte from the ovarian follicle also takes place during late oogenesis. Both oocyte maturation and ovulation have been extensively studied in vertebrates [1-4]. However, other key biological processes that also occur in the ovary during late oogenesis have received far less attention. During this time period, the oocyte acquires not only its ability to resume meiosis, once hormonally stimulated, but also its ability to develop, once fertilized, into a normal embryo. These two processes are referred to as maturational competence acquisition and developmental competence acquisition, respectively. They both rely on events that could occur very early in the oogenetic process. For example, the accumulation of maternally-inherited mRNAs into the oocyte that can occur very early during oogenesis is known to support embryonic development until the activation of the zygotic genome [2,5,6]. It is also noteworthy that oocyte-somatic cell interactions and communications participate, at least in mammals, in the acquisition of the ability of the oocyte to resume meiosis, to be ovulated, to be fertilized, and to subsequently develop into a normal embryo [7]. Nevertheless, maturational and developmental competence acquisitions remain poorly understood, especially in non-mammalian vertebrates in which available data remain scarce. This problem is further complicated by the large variety of ovarian development types found among those species. In teleosts for instance, the dynamics of follicular recruitment can be extremely variable in comparison to what is seen in mammals [8]. It should however be stressed that amphibians and teleosts share a common ovarian follicular structure that is also very different from the mammalian follicle in which an antrum is formed during late folliculogenesis [2]. This common feature could suggest that conserved mechanisms may exist among these two non-mammalian vertebrate groups. In an attempt to identify conserved molecular mechanisms underlying oocyte development, the present study was designed to identify common genes that are differentially expressed by the ovarian follicles during late oogenesis in a teleost fish and an amphibian. The rainbow trout (*Oncorhynchus mykiss*) and the south-African clawed toad (*Xenopus laevis*) were selected because both species have significant genomic resources and a large background in oocyte maturation studies [9,10]. Moreover, *Xenopus *ovarian development is asynchronous and follicles of all stages (I-VI) can be found in the same ovary at any time [11]. While this type of ovarian development can also be found in numerous teleosts, the rainbow trout has, in contrast, a group-synchronous ovarian development type and all follicles of a single clutch develop simultaneously once a year [12]. Thus, we aimed at identifying molecular mechanisms that would be conserved among the two studied vertebrate groups despite existing differences in ovarian development types. Indeed, it should be stressed that amphibians and teleosts share common mechanisms such as the predominant role of progesterone or a progestin, the maturation-inducing steroid, in the induction of meiosis resumption [10,13]. To achieve our goal, differentially expressed genes were selected from an existing microarray study previously carried out in rainbow trout [14] and subsequently studied in both species using quantitative PCR (QPCR). The comparative QPCR-based analysis was carried out during late oogenesis at successive steps of maturational competence acquisition in both species. Finally, the nature of some common molecular mechanisms was highlighted through a deeper analysis of specific genes encoding proteins with known or suspected functions in steroidogenesis.

## Results

### Candidate genes selection

**Figure 1:**
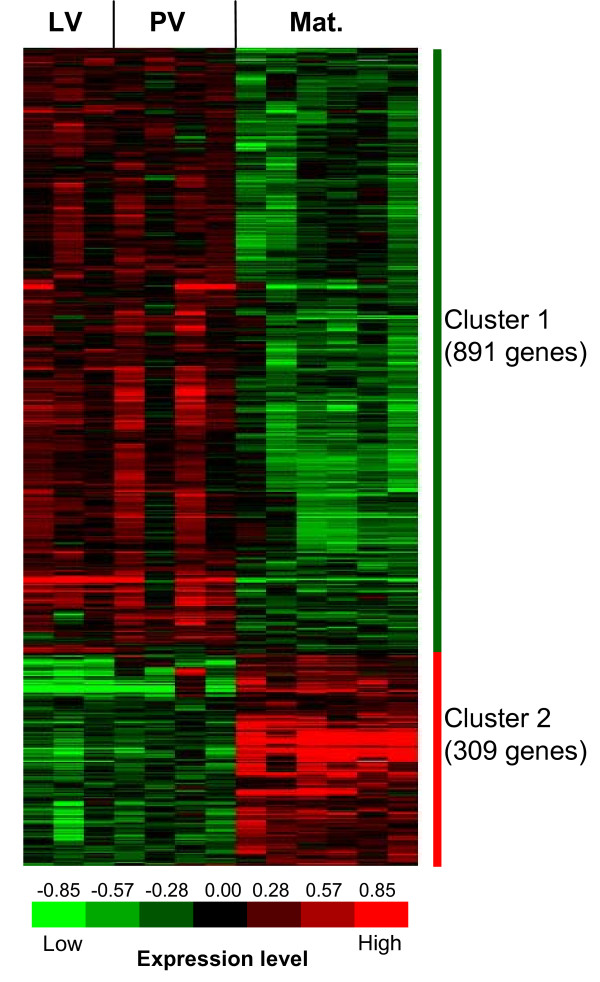
**Gene expression profiling during rainbow trout late oogenesis **Supervised average linkage clustering analysis of 1200 clones in rainbow trout ovary during late vitellogenesis (LV, n = 3), post-vitellogenesis (PV, n = 4) and during maturation (Mat., n = 6). Each row represents a gene and each column an ovarian RNA sample. The 13 samples are supervised according to the natural time-course of oogenesis. Data were log2-transformed and median-centered prior to the clustering analysis. For each gene the expression level within sample set is indicated using a color density scale. Red and green are used for over- and under-expression respectively, while black is used for median expression.

In a previous study, a microarray analysis was carried out during rainbow trout late oogenesis [14] and data subsequently deposited in Gene Expression Omnibus database (GEO Series accession number GSE4871). This existing dataset was reanalyzed and used to select candidate genes for the QPCR-based comparative analysis carried out hereafter. A Significance Analysis of Microarray (SAM) procedure with a false discovery rate (FDR) of 5% was performed in the present study and resulted in the identification of 1200 differentially expressed genes (Fig. 1A). The clustering analysis of these 1200 clones resulted in the identification of two major clusters of genes. The first cluster contained 891 clones that were down-regulated throughout post-vitellogenesis and oocyte maturation. In contrast, the second cluster contained 309 clones that were up-regulated during oocyte maturation. Among the 1200 differentially expressed genes, 88% were annotated with at least one GO biological process term (Table 1). Among the most enriched biological processes associated with cluster 1 genes, several ones appeared to be involved in DNA/RNA metabolism and post-translational modifications [see Additional file 1]. When considering cluster 2 genes, the enrichment analysis brought up biological processes involved in the regulation of transcription, cell cycle progression, cell adhesion and cell communication [see Additional file 2].

**Table 1:** Number of Gene Ontology (GO) functional annotation terms associated with differentially expressed genes using DAVID software

	Number of differentially expressed genes	GO annotated genes	genes with associated GO biological process
**Total**	1200	1059 (88.25%)	1056 (88%)
**Cluster 1**	891	764 (85.75%)	759 (85.18%)
**Cluster 2**	309	304 (98.38%)	297 (96.12%)

The number of differentially expressed genes, the number and percentage of genes associated with at least one GO functional annotation term, and the number and percentage of genes associated with at least one GO biological process term are indicated. Clusters 1 and 2 correspond to the gene clusters presented in fig. 1.

Together, these functions are consistent with the various physiological processes undergone by the follicle-enclosed oocyte during the preovulatory period (i.e. ovulation, fertilization and embryo development) [1].

Twenty-three genes exhibiting at least a two-fold change in expression between late/post-vitellogenesis and oocyte maturation were selected among the differentially expressed genes (Table 2) for the QPCR-based comparative analysis. In addition, three genes (11β 3-hydroxysteroid dehydrogenase *hsd11b3*, the connexin *cx32.2 *and the transcription factor *etv5*) that exhibited a lower differential expression were also kept for further analysis based on their putative role in oocyte maturational and developmental competence acquisition [15,16]. A total of 26 genes was thus kept for further QPCR-based comparative analysis (Table 2). *Xenopus *sequences were identified by a reciprocal best blast hit strategy using the rainbow trout sequence as a query. Accession number and symbol of *Xenopus *genes are also shown in table 2. These genes are involved in different cellular functions such as metabolic processes, trafficking, proteolysis, transcription, cell cycle, and post-translational protein modifications.

**Table 2:** Genes assayed by QPCR in ovarian samples from *Oncorhynchus mykiss *and from *Xenopus laevis*

	*-------------------Oncorhynchus mykiss-----------------*								*----------------------Xenopus laevis-------------------*				
**target genes**	**symbol**	**GenBank#**	**LV/PV**	**LV/MAT**	**PV/MAT**	**LV/PV**	**LV/MAT**	**PV/MAT**	**symbol**	**GenBank#**	**IV/VI**	**IV/MAT**	**VI/MAT**
			**------microarray ratio----**			**--------QPCR ratio--------**					**--------QPCR ratio--------**		

*Aromatase	*cyp19a1a*	BX083177	2.58	25	9.67	2.6	182	76	*P450arom-A*	NM_001085653	313	1237	3.95
*Cytochrome P450 17A1	*cyp17a1*	BX072662	0.84	2.46	2.92	1.15	4.7	4.5	*cyp17a1*	AF325435	2.41	3.77	1.56
* RNA-binding region-containing protein 39	*rbm39a*	BX081888	1.77	3.06	1.73	0.59	0.52	0.99	*rbm39*	BC077813	1.68	2.25	1.34
* A disintegrin and metalloproteinase domain 8	*adam8a*	BX871415	0.50	0.23	0.45	1.18	0.10	0.18					
A disintegrin and metalloproteinase domain 13									*adam13*	NM_001087445	2.28	2.94	1.29
* A disintegrin and metalloproteinase domain 22	*adam22*	CA363158	1.00	0.42	0.42	0.47	0.14	0.30					
A disintegrin and metalloproteinase domain 11									*mdc11b*	NM_001087444	1.41	1.65	1.17
* Protein kinase C delta type	*prkcd*	CA372310	1.02	0.52	0.51	0.77	0.09	0.12	*pkc-delta2*	NM_001090993	1.43	2.19	1.52
* Serum/glucocorticoid related kinase 2	*sgk2*	CA387850	0.99	0.24	0.24	0.01	0.0007	0.08	*sgk1a*	NM_001090340	2.97	1.72	0.58
* Serine protease 23	*prss23*	BX087643	0.53	0.26	0.49	0.21	0.07	0.35	*prss23*	CD255705	2.79	7.85	2.82
* Regulator of G-protein signaling 18	*rgs18*	BX876662	1.04	0.42	0.40	0.94	0.98	1.18	*rgs18*	NM_001092321	1.95	2.17	1.11
* Forkhead box O5	*foxo5*	BX885992	0.91	0.32	0.35	0.55	0.10	0.19					
Forkhead box O3									*foxo3*	NM_001092949	1.94	1.85	0.95
* **Cytidine monophosphate-N-acetylneuraminic acid hydroxylase**	*cmah*	BX878414	1.28	5.88	4.58	1.26	10.57	9.26	*cmah*	NM_001086828	7.26	6.16	0.85
*Steroidogenic acute regulatory protein	*star*	BX079021	0.81	0.34	0.41	0.56	0.20	0.35	*star-A*	AF220437	0.23	0.02	0.10
* Growth arrest and DNA-damage-inducible protein beta	*gadd45bii*	CA363171	0.83	0.36	0.43	0.59	0.14	0.26	*gadd45b*	DT070441	2.41	2.89	1.20
*Apolipoprotein CI	*apoc1*	CA353171	0.27	0.14	0.53	0.26	0.13	0.52	*apoc1*	DQ096911	0.23	0.40	1.75
*11-beta 3-hydroxysteroid dehydrogenase	*hsd11b3*	CA348069	0.83	1.2	1.45	1.29	3.69	2.97	*hsd11b3*	BC106472	2.18	3.09	1.41
* Receptor-type tyrosine-protein phosphatase F	*ptprf*	CA360891	0.68	0.38	0.56	0.69	0.28	0.43	*xptp-d*	NM_001090381	2.71	8.97	3.31
* Dystrophin	*dmd*	CA377239	0.83	0.11	0.13	0.53	0.55	0.84	*dmd*	X99700	2.11	2.38	1.13
* Cyclin L1	*ccnl1*	BX863114	0.79	0.55	0.70	1.01	0.65	0.64	*ccnl1*	BC073707	1.40	1.66	1.19
*Transferrin receptor protein 1	*tfr1*	BX860777	1.36	3.07	2.26	1.47	3.41	2.50	*tfr1*	CA790915	1.98	7.12	3.58
* C-ETS-2 protein	*ets2*	CA368141	0.99	0.39	0.39	1.11	0.36	0.34	*ets2b*	X52635	1.40	1.96	1.40
* ETS translocation variant 5	*etv5*	BX870637	0.98	0.71	0.74	0.59	0.62	1.18					
XER81									*xer81*	AF057565	1.38	1.69	1.22
* Gap junction Connexin-32.2 protein	*cx32.2*	BX082081	0.96	1.2	1.25	0.17	0.13	0.82	*cx32*	DQ096928	1.28	2.45	1.91
* Tight junction protein ZO-1	*tjp1*	BX872029	0.87	0.48	0.55	0.85	0.45	0.54	*tjp1*	BC088825	2.36	2.84	1.21
* Claudin-11	*cldn11*	BX301535	0.77	0.37	0.48	0.43	0.16	0.39	*cldn11*	CB591807.1	1.90	2.66	1.40
*Tescalcin	*tesc*	BX877446	0.71	2.34	3.27	0.84	13.19	13.90	*tesc*	NM_001095029	2.44	2.34	0.96
* 7-dehydrocholesterol reductase	*dhcr7*	BX884545	0.87	0.48	0.55	1.17	0.26	0.25	*dhcr7*	NM_001087087	1.96	1.79	0.91

For each gene, official name, official symbol, GenBank accession number, and expression ratio among studied stages deduced from microarray and QPCR analyses are indicated. Target gene names are bolded when their expression profile during late oogenesis is similar in both species. Thirteen rainbow trout females were used for the transcriptomic analysis (FV, n = 3; PV, n = 4; females; Mat., n = 6) [14], while 28 females (FV, n = 6; PV, n = 14 females; Mat., n = 8) were used for QPCR, including the females used for the transcriptomic analysis.

### QPCR-based comparative analysis during late oogenesis

**Figure 2:**
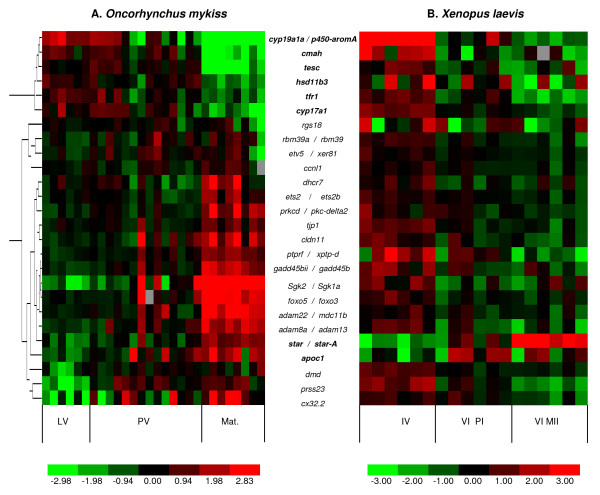
**Expression of 26 genes assessed by real-time PCR in *Oncorhynchus mykiss *and *Xenopus laevis *ovarian samples**. QPCR analysis of the 26 candidate genes in rainbow trout (A) ovary during late vitellogenesis (LV, n = 6), post-vitellogenesis (PV, n = 14) and during maturation (Mat., n = 8), and in *Xenopus *(B) ovarian follicles at stage IV, stage VI and after oocyte maturation (metaphase-II arrested oocytes) from six adult females. Data were normalized to the abundance of 18S, log2-transformed, and median-centered prior to the clustering analysis. The expression data sets have been supervised according to oogenesis stage. Genes with similar expression profiles in both species are bolded. The dendrograms on the left represent correlation distances between the profiles of studied trout genes. For each gene the expression level within sample set is indicated using a color density scale. Red and green are used for over- and under-expression respectively, while black is used for median expression (grey boxes, not determined).

In rainbow trout, a differential expression was demonstrated for 24 genes out of 26 (Fig. 2). For those 24 genes, the expression ratios calculated from QPCR and microarray data are consistent even if some limited differences exist, mostly due to the underestimation of highest differential expression by nylon array technology (Table 2). Six genes belonging to cluster 1 (*cyp19a1a, cyp17a1, tescalcin, tfr1, cmah, hsd11b3*) showed a decreased expression during maturation. Eighteen genes belonging to cluster 2 exhibited an increased mRNA expression throughout late oogenesis. These genes are mostly related to proteolysis, regulation of transcription, cell cycle and cellular junctions. Contrarily to the results of the microarray analysis, two genes (*rbm39a *and *rgs18*) showed a stable expression profile throughout late oogenesis. In *Xenopus*, 24 among the 26 assayed genes were down-regulated during late vitellogenesis and/or maturation whereas only 2 genes (the apolipoprotein *apoC1*, and the STeroidogenic Acute Regulatory protein *star-A*) showed a sharp increase of mRNA expression during late vitellogenesis or at the time of oocyte maturation, respectively (Fig. 2). In summary, eight genes shared similar expression profiles in both rainbow trout and *Xenopus *during late oogenesis. Among these genes, six were down-regulated during oocyte maturation (*cyp19a1a, cyp17a1, tescalcin, tfr1, cmah, hsd11b3*) while two exhibited an opposite pattern (*apoc1, star*).

### Characterization of genes with known or suspected functions in steroidogenesis

Among the eight genes exhibiting similar expression profiles in both species, four genes encode proteins with known or suspected functions in steroidogenesis and were further studied here. In addition, the apolipoprotein C1 (apoC1) may also be linked to steroidogenesis as the cholesterol necessary for ovarian steroidogenesis may come either from *de novo *synthesis or from lipoprotein cholesterol uptake. *ApoC1 *was therefore selected for further analysis. In vertebrates, including rainbow trout and *Xenopus*, the orthology relationships of cytochrome P450 19A1 (*cyp19a1*) and STeroidogenic Acute Regulatory protein (*star*) are well characterized [17-20] (although *star-A *is the *Xenopus *official symbol, we will use *star *hereafter for both trout and *Xenopus *genes for clarity reason). In contrast, *Xenopus *orthologs of *cyp17a1, hsd11b3 *and *apoC1 *had not been previously identified clearly. We thus performed specific phylogenetic analyses that are reported in additional files 3, 4 and 5 for *cyp17a1, hsd11b3 *and *apoC1 *respectively, to demonstrate orthology relationships between rainbow trout and *Xenopus *sequences.

**Figure 3:**
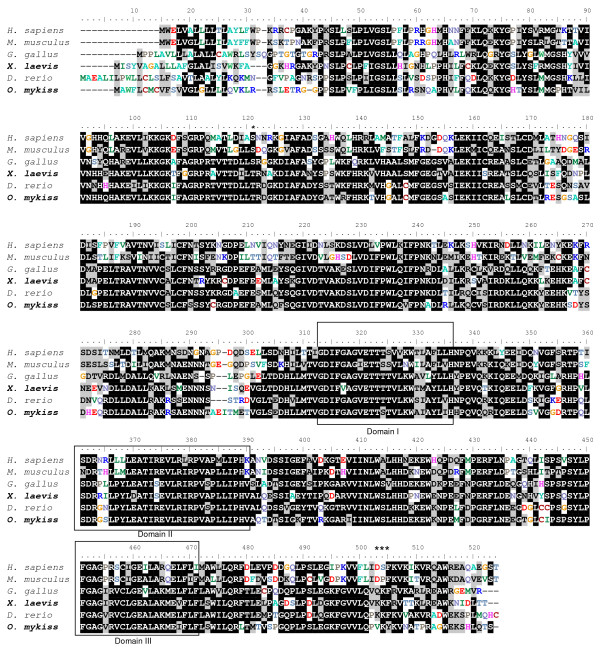
**Cyp17a1 amino acid sequence alignments among vertebrates**. Amino acid sequence alignments between human CYP17A1 (ENSG00000148795, *H. sapiens*), mouse Cyp17a1 (NP_031835.3, *M. musculus*), chicken CYP17A1 (ENSGALG00000008121 peptide ENSGALP00000032532, *G. gallus*), clawed toad Cyp17a1 (AAG42003, *X. laevis*), zebrafish Cyp17a1 (AAI62669.1, *D. rerio*) and rainbow trout Cyp17a1 (NP_001118219.1, *O. mykiss*). Multiple amino acid sequence alignments were constructed using ClustalW software. The conserved domains previously identified are indicated: domain I (heme-binding domain [22]); domain II (putative steroid-binding domain [23]); domain III (CYP17 specific domain [21]). The amino acids that have been evidenced as essential for human CYP17 activity are indicated with asterisks (serine 106, aspartic acid 487, serine 488 and phenylalanine 489) [24,25].

The rainbow trout and *Xenopus *Cyp17a1 protein sequences exhibit 47% overall sequence identity with the cognate human protein. The rainbow trout Cyp17a1 protein sequence exhibits 61% sequence identity with cyp17a1 *Xenopus *sequence (Fig. 3). Three conserved domains have been characterized [21]. The domain I is composed of a heme-binding domain [22] and displays 71% of identity between human and trout sequences, and 79% of identity between trout and *Xenopus *sequences. The domain II is a conserved tridecapeptide, with a putative steroid-binding domain [23]. This domain is 77% identical between human and trout sequences and 87% between trout and *Xenopus *sequences. The domain III has been described as a CYP17 specific domain [21] and shows 62% of identity between human and trout sequences and 90% of identity between trout and *Xenopus *sequences. Finally, a mutation of the human ser-106 or a deletion of the three amino acids asp-487, ser-488 and phe-489 abrogate CYP17 activity [24,25]. Interestingly, these amino-acids are poorly conserved in non-mammalian vertebrates.

**Figure 4:**
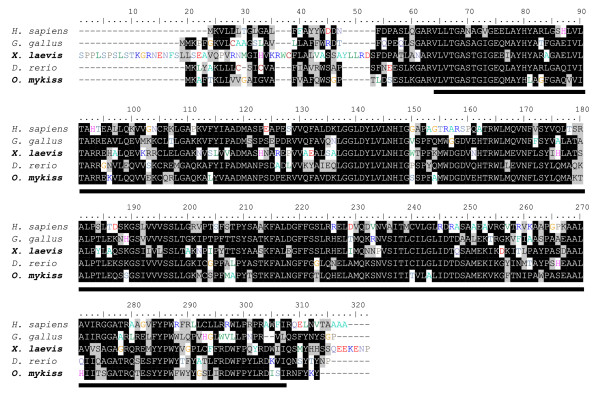
**hsd11b3 amino acid sequence alignments among vertebrates**. Amino acid sequence alignments between human HSD11B3 (ENSP00000340436, *H. sapiens*), chicken HSD11B3 (NP_001001201.1, *G. gallus*), clawed toad hsd11b3 (BC106472, *X. laevis*), zebrafish hsd11b3 (ENSDARG00000004562, *D. rerio*) and rainbow trout hsd11b3 (CA348069, *O. mykiss*). Multiple amino acid sequence alignments were constructed using ClustalW software. The superfamily Rossmann-fold NAD(P)H/NAD(P)(+) binding domain is underlined.

Fish Hsd11b3 is orthologous to *Xenopus*, chicken and human hsd11b3, also referred as hsd11b1-like (Additional file 4). Rainbow trout Hsd11b3 shares 49% and 47% identity with human and *Xenopus *proteins respectively (Fig. 4) while *Xenopus *and human sequences are 42% identical. Hsd11b proteins possess a superfamily Rossmann-fold NAD(P)H/NAD(P)(+) binding domains composed of a glucose/ribitol dehydrogenase domain and a short chain dehydrogenase domain.

**Figure 5:**
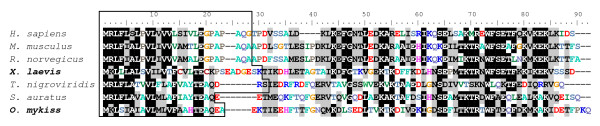
**apoC1 amino acid sequence alignments among vertebrates**. Amino acid sequence alignments between human APOC-I (NP_001636.1, *H. sapiens*), mouse APOC-I (NP_031495.1, M. *musculus*), rat APOC-I (NP-036956.1, *R. norvegicus*), the clawed toad apoC1 (CF547196, *X. laevis*), tetraodon apoC1 (CAF95503, *T. nigroviridis*), the gilthead seabream apoc1 (AAT45249.1, *S. auratus*) and the deduced amino acid sequence of rainbow trout apoC1 (CA353171, *O. mykiss*). Multiple amino acid sequence alignments were constructed using ClustalW software. The signal peptide is indicated.

Finally, our phylogenetic analysis clearly demonstrated the orthology relationship of rainbow trout and *Xenopus *ApoC1 genes [see Additional file 5]. At the amino acid level, rainbow trout and *Xenopus *sequences share 36% of identity. The trout sequence is 23% identical to the human protein, while *Xenopus *sequence is 29% identical to the human protein (Fig. 5).

### Ovarian expression and tissue distribution of *star *in rainbow trout and *Xenopus*

**Figure 6:**
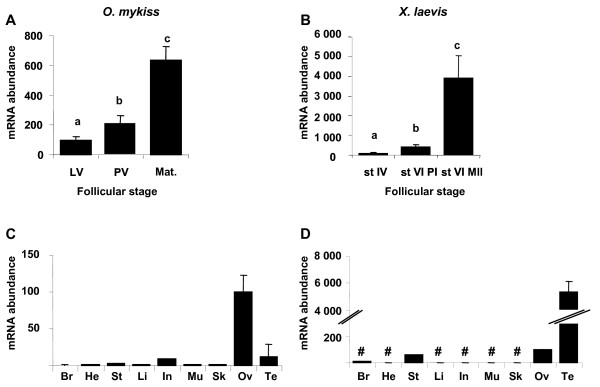
***star *expression profiles during late oogenesis and tissue expression profiles**. Expression profiles of *star *in rainbow trout ovary sampled from females during late vitellogenesis (LV, n = 6), post-vitellogenesis (PV, n = 14) and during maturation (Mat., n = 8) (A), in *Xenopus laevis *ovarian follicles sampled from six females, at stage IV, stage VI and after *in vitro *maturation (st VI MII) (B). Expression of *star *mRNA in rainbow trout tissues: brain (Br), heart (He), stomach (St), liver (Li), intestine (In), muscle (Mu), skin (Sk), post-vitellogenic ovary (Ov), and testis (Te) (C) and in *Xenopus laevis *tissues: brain (Br), heart (He), stomach (St), liver (Li), intestine (In), muscle (Mu), skin (Sk), ovary (Ov), testis (Te). Data were normalized to the abundance of 18S. Mean and SEM are shown. Bars sharing the same letter(s) are not significantly different (p > 0.05). In tissue, expression levels which are not significantly different from background signal are indicated with #.

*Star *mRNA expression shows a progressive increase during late and/or post-vitellogenesis and a sharp increase during maturation in both species (Fig. 6A and 6B). In rainbow trout, *star *mRNA abundance is two times higher during post-vitellogenesis than during late vitellogenesis and six times higher during maturation than during late vitellogenesis. In *Xenopus laevis, star *mRNA abundance is four times higher at prophase I of stage VI and 40 times higher at metaphase II of stage VI, when compared to stage IV follicles. Tissue analysis revealed a predominant expression in gonads of trout and *Xenopus *(Fig. 6C and 6D). The expression of *star *in *Xenopus *testis was 60 times higher than in ovary. In contrast, expression of *star *in trout testis was six times lower than in ovary. Low expression levels were also evidenced in trout intestine and to a less extent in other tissues. *Star *mRNA expression was also detected in *Xenopus *stomach.

### Ovarian expression and tissue distribution of aromatase in rainbow trout and *Xenopus*

**Figure 7:**
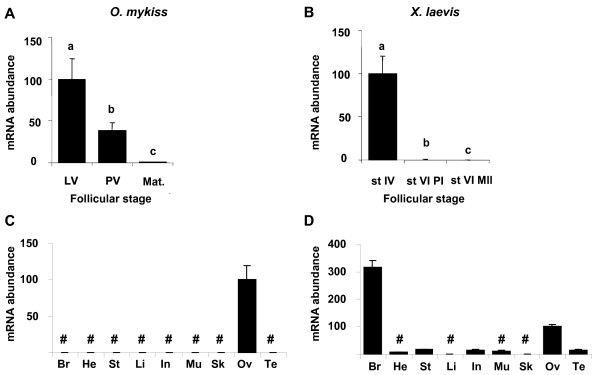
**aromatase expression profiles during late oogenesis and tissue expression profiles**. Expression profiles of aromatase (*cyp19a1a *for trout and *p450arom-A *for *Xenopus*) during late oogenesis and in various tissues. Experiments were conducted in parallel for all genes. See fig. 6 legend for details.

The cytochrome P450 aromatase (*cyp19a1a *for trout and *p450-arom-A *for *Xenopus*) expression shows a dramatic decrease during late oogenesis in both species (Fig. 7A and 7B). The transcript thus becomes barely detectable in matured follicles of both species. Tissue expression analysis confirms an ovarian-specific expression of the gonad gene (*cyp19a1a*) for trout (Fig. 7C) with no transcript evidenced in brain. When using primers that do not discriminate ovarian and cerebral aromatase mRNAs, we detected expression of aromatase mainly in *Xenopus *brain and ovary (Fig. 7D). The higher expression was detected in the brain and a weaker expression of aromatase mRNA was detected in testis, stomach and intestine.

### Ovarian expression and tissue distribution of *cyp17a1 *in rainbow trout and *Xenopus*

**Figure 8:**
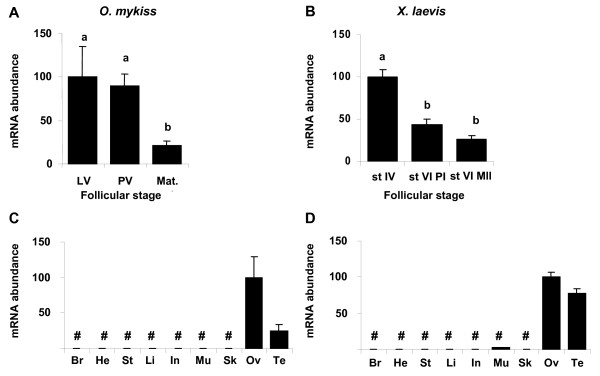
***cyp17a1 *expression profiles during late oogenesis and tissue expression profiles**. Expression profiles of *cyp17a1 *during late oogenesis and in various tissues in rainbow trout and *Xenopus*. Experiments were conducted in parallel for all genes. See fig. 6 legend for details.

*cyp17a1 *expression decreased from late and/or post-vitellogenesis to the completion of oocyte maturation in both species, but in a slightly different manner (Fig. 8A and 8B). In trout, a significant four time reduction was evidenced only during maturation even if the decrease was initiated earlier, by the end of vitellogenesis. On the other hand, *Xenopus cyp17a1 *expression decreased significantly during late/post-vitellogenesis as shown by the two time mRNA down-expression in oocytes from stage IV to stage VI. Then, the decrease of *cyp17a1 *was less important and not significantly different in follicles during oocyte progression from prophase I to metaphase II of meiosis. Tissue analyses showed that its expression was restricted to gonads in both species (Fig. 8C and 8D).

### Ovarian expression and tissue distribution of *hsd11b3 *in rainbow trout and *Xenopus*

**Figure 9:**
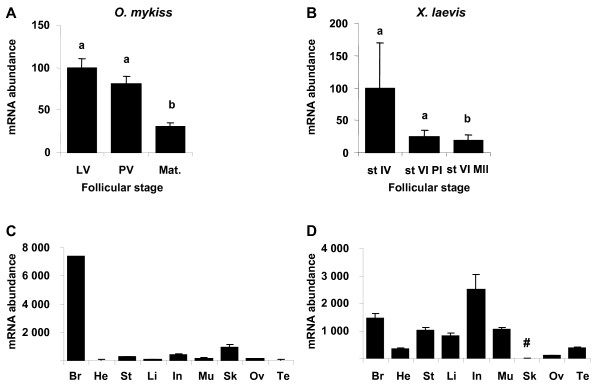
***hsd11b3 *expression profiles during late oogenesis and tissue expression profiles**. Expression profiles of *hsd11b3 *for trout and *Xenopus*. Experiments were conducted in parallel for all genes. See fig. 6 legend for details.

*hsd11b3 *is nearly three-times less expressed during maturation than during late vitellogenesis in both trout and *Xenopus *(Fig. 9A and 9B). In trout, *hsd11b3 *expression significantly decreased at the time of oocyte maturation like in *Xenopus*. In the amphibian, the transcript reduction was already visible during late vitellogenesis but was not significant because of variable *hsd11b3 *mRNA levels measured in *Xenopus *stage IV follicles from different females. In contrast to the other three gene candidates analyzed above, *hsd11b3 *was expressed in a more ubiquitous fashion (Fig. 9C and 9D). In trout and *Xenopus, hsd11b3 *was expressed in brain and intestine. Trout skin mRNA abundance was nine times higher than in the ovary, while no expression of *hsd11b3 *was detected in *Xenopus *skin. Moreover no expression of *hsd11b3 *was detected in trout stomach, muscle, liver and heart while *hsd11b3 *was expressed in these tissues in *Xenopus*.

### Ovarian expression and tissue distribution of *apoC1 *in rainbow trout and *Xenopus*

**Figure 10:**
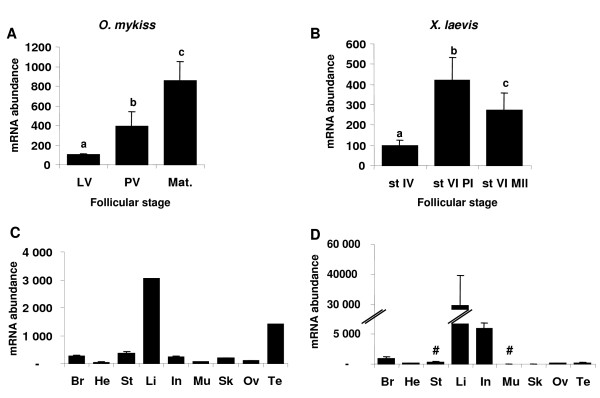
***apoC1 *expression profiles during late oogenesis and tissue expression profiles**. Expression profiles of *apoC1 *for trout and *Xenopus*. Experiments were conducted in parallel for all genes. See fig. 6 legend for details.

ApoC1 mRNA level is four times more expressed during post-vitellogenesis than during late vitellogenesis in trout and *Xenopus *(Fig. 10A and 10B). Moreover, *apoC1 *expression increases dramatically during oocyte maturation in trout, while its expression slightly decreases during maturation in *Xenopus *but remains nearly three times higher than during late vitellogenesis. In trout and *Xenopus, apoC1 *is mainly expressed by the liver and to a less extent, in the intestine, brain, stomach, ovary and testis (Fig. 10C and 10D).

## Discussion

### Identification of differentially expressed orthologous genes in both species

Among the twenty-six genes studied in the QPCR-based comparative analysis, eight genes had similar expression profiles throughout late oogenesis in both rainbow trout and *Xenopus*. These results indicate that our strategy which consisted in reanalyzing a transcriptomic study in one species followed by a subsequent QPCR study in both species is relevant for the identification of orthologous genes that possibly participate in conserved molecular mechanisms among the two species. Among the eight genes exhibiting similar profiles in both species, five genes could be linked directly or indirectly to steroidogenesis, thus pointing out steroidogenic-related mechanisms as being possibly well conserved among fish and amphibians. Although gene expression of *star, cyp19a1a *and *cyp17a1 *have already been described in trout during late oogenesis [14,26,27], none of these genes have been described at the mRNA level during this period in *Xenopus*. Moreover no expression data regarding *Hsd11b3 *and *apoC1 *are available in any of the studied species. Due to the predominant role of sex steroids in the control of the reproductive process in teleosts and amphibians [2,10], these 5 genes were further studied in the present study.

While our approach has been successful, it should however be pointed out that a significant number of genes over-expressed during maturation in trout did not exhibit a similar pattern in *Xenopus*. These differences could be due to species-specific differences or even correspond to non-conserved mechanisms. Another possible explanation for the discrepancy between trout and *Xenopus *is that in the present study, trout oocyte maturation occurred naturally *in vivo*. In contrast, *Xenopus *oocyte maturation was triggered *in vitro *by a 15-h incubation of isolated fully-grown follicles in medium supplemented with human chorionic gonadotropin (hCG). It is thus possible that the *in vitro *conditions did not trigger all the mechanisms that are naturally occurring in the preovulatory follicle, especially those involved in ovulation. Indeed, even if *Xenopus *injection of heterologous gonadotropin can lead to *in vivo Xenopus *oocyte maturation and ovulation [28], these processes can sometimes be uncoupled *in vitro *[29]. This could explain, in part, our results.

Finally, in order to identify *Xenopus *genes related to the trout candidates we used, as a high throughput approach, a best blast hit strategy. The orthology relationships between trout and *Xenopus *cognate proteins were then validated only for the five candidates that we subsequently analyzed. Thus, we cannot totally rule out that a part of the discrepancy between trout and *Xenopus *profiles could be due to a misidentification of true orthologs in *Xenopus*. This is especially true for *adam8a, adam22, foxo5 *and *etv5 *genes that will require detailed phylogenic analyses.

### Candidate gene analysis

#### Star. *STeroidogenic Acute Regulatory protein*

(Star) is involved in cholesterol shuttling across the mitochondrial membrane and its synthesis appears to be a limiting step in steroidogenesis [30-34]. In the present study, *star *was shown to be predominantly expressed in rainbow trout and *Xenopus *gonads. These results are in agreement with existing data in various vertebrates including teleosts [19,20,33]. Low expression levels were also detected in trout intestine and in *Xenopus *stomach. Although *star *expression has already been reported in rainbow trout intestine [19], no expression has been evidenced in digestive tract in any other species. While the over-expression during follicular maturation was previously documented in rainbow trout [19,26], we showed for the first time that *star *is also over-expressed during hCG-induced oocyte maturation in *Xenopus*. Interestingly, *star *expression is induced in response to gonadotropin stimulation in mammals and birds [30,35-38]. As oocyte maturation is induced in response to LH (Luteinizing Hormone) stimulation in fish and amphibians [2], it is likely that the strong *star *mRNA over-expression reported here in trout and *Xenopus *is also triggered by LH-mediated signaling pathway(s). In rainbow trout, it is also noteworthy that the circulating levels of the maturation-inducing steroid (MIS) detected during the preovulatory period [39] increase concomitantly with *star *mRNA levels. Likewise, in *Xenopus laevis*, progesterone and testosterone secretions are more important in stage VI follicles compared to stage IV follicles in agreement with *star *increased expression observed in this study [40]. Together, these results point out the over expression of *star *by gonadotropin during oocyte maturation as a possible conserved mechanism among non-mammalian vertebrates and possibly all vertebrates. The nature of the corresponding protein emphasizes the importance of steroidogenesis in the control of late oogenesis in non-mammalian vertebrates.

#### Aromatase

Aromatase is an enzyme (CYP19; EC 1.14.14.1) that converts androgens to estrogens. In most species including humans, chicken, *Xenopus *and a cartilaginous fish, a single gene has been isolated [41-44]. In contrast, in most teleosts, two genes, *cyp19a1a *(also referred as *cyp19a *or *cyp19a1*) and *cyp19a1b *(also referred as *cyp19b *or *cyp19a2*), encode distinct proteins predominantly expressed in the ovary and the brain, respectively [17,45-47]. In the present study, we show that *cyp19a1a *is only expressed in trout ovary, while *p450arom-A *is expressed mainly in *Xenopus *gonads and brain. The lack of *cyp19a1a *expression in rainbow trout brain is somewhat surprising as *cyp19a1a *has previously been detected in brain of various fish species [46-49]. The lack of expression of *cyp19a1a *in trout testis is, in contrast, in agreement with a previous study carried out in zebrafish [46]. In our study, a weak expression of aromatase was detected in *Xenopus *testis. This observation is consistent with a previous study reporting a weak expression but no aromatase activity in *Xenopus laevis *adult testis [50]. In *Xenopus*, we evidenced a very low expression in intestine and stomach, thus corroborating studies in human fetus which showed an aromatase expression in intestine [51,52].

The cytochrome P450 aromatase expression decreases dramatically during late oogenesis in both *Xenopus *and trout resulting in a barely detectable aromatase expression during maturation. Previous studies already indicated a decrease of aromatase expression [14,53-57] and activity [58] as well as a reduction of circulating E2 levels [26,27,39,59] throughout late oogenesis in several fish species. However, we report here for the first time that aromatase transcript expression decreases dramatically in *Xenopus *post-vitellogenic, immature, follicles throughout late oogenesis. This observation is consistent with the decrease of aromatase and E2 production by the ovarian follicle during the post-vitellogenic period [40,60]. Together, these observations suggest that the drop of aromatase mRNA expression in the late oogenetic follicle is possibly a conserved molecular mechanism among non-mammalian vertebrates. In addition, existing data on the inhibition of oocyte maturation by E2 obtained in fish [61] as well as in another amphibian, *Rana pipiens *[62,63] suggest that this mechanism of inhibition of precocious meiosis resumption could contribute to oocyte maturational competence acquisition in non-mammalian vertebrates.

#### Cyp17a1

The cytochrome P450 17A1, for which the official symbol is *cyp17a1 *is a member of the large superfamily of cytochrome P450. This enzyme (CYP17A1; EC = 1.14.99.9) acts as a 17α-hydroxylase and a 17-20-lyase. The 17α-hydroxylase activity converts progesterone and pregnenolone to 17α-hydroxyprogesterone and 17α-hydroxypregnenolone, respectively while the 17,20-lyase activity converts 17α-hydroxypregnenolone to dehydroepiandrosterone (DHEA) and 17α-hydroxyprogesterone to androstenedione. DHEA and androstenedione are precursors of testosterone and estrogen synthesis while 17α-hydroxyprogesterone is a precursor of different progestins and cortisol. In teleosts, two genes have been identified [64]: *Cyp17a1 *(previously referred as *P450-I*) and *cyp17a2 *(previously referred as *P450-II*). *Cyp17a1 *encodes a protein exhibiting both activities, as in other vertebrates, whereas *cyp17a2 *encodes a protein lacking 17,20 lyase activity.

*Cyp17a1 *is expressed in the gonads of both species, consistently with previous data in mammals [65], birds [66], and fish [67,68], including rainbow trout [26,27,69,70] but reported here for the first time in an amphibian species. In a previous study, a strong mRNA expression was also reported in rainbow trout kidney [70]. These authors also detected a low expression in various tissues using semi-quantitative RT-PCR that we were not able to confirm in the present work using QPCR. In both species, ovarian mRNA levels decrease dramatically throughout late oogenesis. In rainbow trout, the *cyp17a1 *profile was previously been documented [26,27] and found to be consistent with previous Northern blot data indicating that *cyp17a1 *was abundant in the post-vitellogenic ovary as a result of an increase of expression that occurred during vitellogenesis [69]. Moreover, it has been shown in tilapia that *cyp17a1 *expression in granulosa cells decreases during late oogenesis, while *cyp17a2 *expression increases [64]. It should be however noticed that *cyp17a1 *expression during late oogenesis can be variable depending on the species [67,71]. Nevertheless, a low *cyp17a1 *mRNA expression was also reported in female fat head minnow close to full sexual maturity [68] and in two catfish populations, *cyp17 *mRNA level was shown to decrease throughout the period of spawning [55]. In rat, both *cyp17a1 *mRNA and protein abundance increase throughout folliculogenesis and subsequently decrease after hCG stimulation of preovulatory follicles [72]. Similarly, *cyp17a1 *mRNA expression decreased after LH surge in bovine preovulatory follicles [73]. Consistent with these observations was the report of a decrease of *cyp17a1 *mRNA expression in follicular layers of the preovulatory chicken follicles [56,74]. Together, existing and present observations suggest that even though species-specificity may exist, the decrease of *cyp17a1 *mRNA expression towards the end of oogenesis may be a well conserved molecular mechanism, possibly related to maturational competence acquisition. Indeed, Cyp17a1 could participate in the shift from E2 to maturation-inducing steroid production observed in fish and amphibians during late oogenesis [40,75]. In addition, Cyp17a1 could also participate in the production of androgens that have been evidenced to play important roles during late oogenesis in both amphibians and fish [76-78].

#### Hsd11b3

The 11beta-hydroxysteroid dehydrogenase isoenzymes HSD11B mainly catalyze the interconversion of active glucocorticoid (cortisol and corticosterone) and inactive 11-keto forms (cortisone and 11-dehydrocorticosterone) [79]. Three proteins have been described in mammals: HSD11B1, HSD11B2 and HSD11B3 (previously known as HSD11B1like or HSD11B1L). Previous analyses have indicated that hsd11b1 was not present in teleost species [80] and could only be found in amphibians, birds and mammals. In fish, Hsd11b2 has been characterized and shown to be expressed in various tissues such as gill, heart, intestine, ovary, testis and skin [79] and studied in more details in the ovary during late oogenesis [79,81]. Interestingly, *hsd11b3 *was phylogenetically characterized [80] but no expression data was available to date in fish and amphibians. In the present study, we show that *hsd11b3 *mRNA is expressed in a large number of tissues but not in *Xenopus *skin while it is expressed mainly in brain, skin and intestine in trout. Thus, rainbow trout *hsd11b3 *mRNA appears to have a more restricted tissue distribution than *hsd11b2 *and tissue expression differs greatly between trout and *Xenopus*. Within the ovary, we evidence a decrease of *hsd11b3 *transcript level throughout late oogenesis in both rainbow trout and *Xenopus *whereas *hsd11b2 *mRNA has been reported to accumulate at the same time in trout female gonad [79,81].

At the functional level, HSD11B2 acts in mammals as a reductase and converts inactive cortisone to active cortisol. In fish, this isoform is also able to convert 11β hydroxy-testosterone into 11keto-testosterone (11-KT), which has been shown to be a major androgen steroid in fish [82]. In contrast, mammalian HSD11B1 predominantly acts in an opposite way, as likely does HSD11B3 according to the evolutionary history of the protein [80], since no functional study has been reported on this isoform. Likewise, fish Hsd11b3 is likely to act as the mammalian HSD11B1 and/or HSD11B3 (Additional file 4). Together, the expression profiles of *hsd11b2 *[79,81] and *hsd11b3 *(present results) in the rainbow trout preovulatory ovary suggest a combined role of these enzymes in gonad protection against any deleterious effect of stress-induced cortisol. Despite the lack of data on glucocorticoid levels and effects during oogenesis in amphibians, we may also hypothesize a similar role of Hsd11b3 during *Xenopus *late oogenesis. We cannot totally rule out a role of Hsd11b3 in regulating 11 ketotestosterone levels during follicular development and oocyte growth. Indeed, 11KT, besides its well-known action in fish spermatogenesis, has been demonstrated as controlling oocyte growth, likely through lipid accumulation in eel [83] and in atlantic cod [84]. However, this cannot be extended to all teleosts as 11KT circulating levels are low or even undetectable in salmonid females [85-87]. Further investigations including protein expression and enzyme activity measurements are thus required to conclude on the exact physiological role(s) of these 11beta-hydroxysteroid dehydrogenase isoenzymes in ovarian functions during late oogenesis.

#### ApoC1

The apolipoprotein C1 belongs to the family of soluble apolipoproteins involved in cholesterol transport and uptake in vertebrates [88]. Interestingly, *in vitro *studies have also suggested that apoC1 could stimulate lecithin-cholesterol acyltransferase (LCAT) activity [89], thereby increasing the formation of esterified cholesterol as well as estradiol ester. In the present study, the tissue expression analysis reveals that *apoC1 *is primarily expressed in trout and *Xenopus *liver, but also in stomach, intestine and gonad. This result is consistent with previous studies performed in mammals [90-92] and in another teleost, *Hemibarbus mylodon *[93]. Only in the orange-spotted grouper, *apoC1 *could not be detected in the liver but it was nonetheless expressed in gonad and in brain [94].

Within the ovary, we show that *apoC1 *mRNA levels dramatically increase during late oogenesis in trout and *Xenopus*. This result confirms previous findings in trout [14] and in orange-spotted grouper [94]; only Tingaud-sequeira *et al*. recently published in a marine flatfish ovary a different pattern of *apoC1 *expression, where its transcript levels significantly increase solely during follicle atresia following ovulation but not during follicle growth or maturation [95]. This study is also the first report on *apoC1 *expression in an amphibian. In mammals, ApoC1 expression in ovary during follicle development has not been studied to our knowledge but the increase of LCAT activity, correlated with a decrease of estradiol/progesterone ratio in antrum of human growing ovarian follicles [96] may indirectly reflect an increase of apoC1 expression. The function of ApoC1, accumulating during late oogenesis, remains to be elucidated. First, this apolipoprotein may have a role in remaining yolk degradation by the oocyte companion cells during follicle atresia [95] or yolk degradation as a nutrient source for early embryo development [94]. ApoC1 could also be involved in the regulation of steroidogenesis at least in two ways: (i) as an inhibitor of lipoprotein uptake *via *inhibition of lipoprotein binding to their receptors [97]; it may then modulate the ovarian cholesterol uptake during oogenesis; (ii) cholestryl and estradiol esters, formed upon LCAT activation by apoC1 [98], remain a source of bioactive metabolites in the steroid synthesis pathways. Interestingly, estradiol esters may also be involved in antioxydation processes [99]. Further studies will be required to fully understand the role(s) of ApoC1 during ovarian follicle development in vertebrates.

## Conclusions

In the present study, we successfully used a QPCR-based comparative analysis to identify genes that are differentially expressed during both rainbow trout and *Xenopus *late oogenesis. Our observations point out several molecular mechanisms as possibly conserved among non-mammalian vertebrates. As an attempt to further characterize some of these mechanisms, we have characterized the sequence of some candidate proteins and thoroughly studied their mRNA expression in various tissues and during post-vitellogenesis in the two studied species. Our data show that the expression profiles of key steroidogenic enzymes are conserved during late oogenesis in *Xenopus laevis *ovarian follicles in agreement with existing data in rainbow trout. We indeed evidenced a decrease of the mRNA levels of *cyp19a1 *and *cyp17a1 *in both species and for the first time in an amphibian species. These expression patterns are consistent with the shift in steroidogenesis of ovarian follicles observed in both amphibians and teleost fish prior to meiosis resumption and could correspond to an important molecular mechanism of oocyte maturational competence acquisition common to these non-mammalian vertebrate groups. In contrast, the levels of *star *mRNA increase during oocyte maturation in both species. The nature of Star protein further stresses the importance of steroidogenesis in the post-vitellogenic process. The timing of *star *over-expression suggests, however, that Star would not be involved only in maturational competence acquisition but also in further follicular processes (*e.g*. ovulation) possibly through an increase of maturation-inducing steroid (MIS) synthesis. In addition, the decrease in *hsd11b3 *mRNA expression observed in both species throughout late oogenesis raises the question of the physiological role of this protein that will require further attention. Finally, the observed increase in *apoC1 *expression may be related to different mechanisms as steroidogenesis regulation through modulation of cholesterol uptake or through regulation of cholesteryl or estradiol esters synthesis. This increased expression may also be linked to yolk processing in the oocyte during late oogenesis or during early embryo development. The characterization of the function of this gene during late oogenesis requires however further investigation. Finally, other genes not related to steroidogenesis were also shown to have similar expression profiles in both species during late oogenesis. The identity of the proteins points out possibly conserved non-steroidogenic mechanisms that deserve future attention and specific investigations.

Together, our results strongly suggest that comparative transcriptomics is an extremely valuable approach that must be pursued to gain insight in the conserved mechanisms underlying maturational and developmental competence acquisition in non-mammalian vertebrates.

## Methods

### Animals and Tissue Collections

Investigations and animal care were conducted in compliance with French and European regulation on the care and use of laboratory animals. Twenty eight adult rainbow trout females (*Oncorhynchus mykiss*) from an autumn-spawning strain were obtained at different stages of ovarian development from the INRA/PEIMA fish farm (Sizun, France). Ovaries were sampled during late vitellogenesis (LV, 3 or 4 weeks before expected spawning, n = 6), in post-vitellogenesis (PV, before oocyte maturation but during spawning period, n = 14) and during maturation (Mat., n = 8). Thirteen of these twenty eight females had previously been used for transcriptomic analysis [14]. For sampling, fish were deeply anaesthetised in 0,1% (v/v) 2-phenoxyethanol in water and killed. Trout ovaries were then dissected out of the body cavity under sterile conditions.

Ovarian pieces were surgically removed from six *Xenopus *anaesthetised adult females (*Xenopus laevis*), purchased from NASCO (Fort Atkinson, WI, USA). 100 *Xenopus *stage IV (st IV, 800-μm diameter) and 50 stage VI (st VI PI, 1200-μm diameter, in prophase I of meiosis) ovarian follicles [11] were manually isolated from ovarian pieces of each female. In order to obtain follicle-enclosed oocytes in metaphase II of meiosis (st VI MII) from the same females, *in vitro *maturation was performed by incubating 50 immature stage VI follicles for 15 h at room temperature in OR2 buffer (83 mM NaCl, 2.5 mM KCl, 1 mM CaCl_2_, 1 mM MgCl_2_, 1 mM Na_2_HPO_4_, 5 mM HEPES, pH 7.4) supplemented with 40 IU/mL of hCG (Organon, Puteaux, France). After the incubation period, germinal vesicle breakdown (GVBD) was assessed by direct observation under a stereomicroscope of the appearance of a white spot on the animal pole of the follicle-enclosed oocytes.

For tissue collection, three *Xenopus *adult females were sacrificed and different tissues (brain, heart, stomach, liver, intestine, muscle, skin, and ovary) were collected in addition to stage IV and VI follicles. The same tissues were also sampled from three different post-vitellogenic rainbow trout females. Trout testis samples were obtained from four different males at stage II (initiation of spermatogenesis) and stage V (spermiogenesis) whereas *Xenopus *testis samples were obtained from one male. Tissue samples were frozen in liquid nitrogen and stored at -80°C until RNA extraction.

### Microarray data analysis

Microarray data obtained from previous hybridization [14] were further reanalyzed. The rainbow trout generic array has been previously deposited in Gene Expression Omnibus (GEO) database (Platform# GPL 3650; GEO accession GSE4871) [100]. In order to identify differentially expressed genes, a statistical analysis was carried out using Significance Analysis of Microarray (SAM) test [101]. Three 2-by-2 statistical analyses were performed in order to compare each group with the two other ones. For each comparison, 5% of false discovery rate (FDR) was used to statistically discriminate differentially expressed genes. For clustering analysis, data were log2-transformed, median-centered, and an average linkage clustering was performed using CLUSTER software. Clusters were visualized using TREEVIEW software [102]. Gene Ontology (GO) analyses were performed using the NIH-DAVID web-based tool (Database for Annotation, Visualization, and Integrated Discovery) [103-105]. Enrichment analyses were realized using all the annotated genes present on the microarray (8 344 genes) as a reference and using UNIPROT accession IDs as input. Enriched GO biological process terms associated with each gene list were determined using modified Fisher's exact tests. GO terms with a raw p-value lower than 0.05 are displayed [see additional files 1 and 2]. The number of genes associated with each GO term and associated enrichment information (raw p-values and fold enrichment) are indicated for each GO term.

### Data mining and sequence analysis

For all the clones identified as differentially abundant after the SAM analysis (Table 2), the official gene symbol was retrieved [14,106,107] when possible and used in the text, figures and tables. The official *Danio rerio *gene symbol was used for the rainbow trout. Rainbow trout cDNA clones and corresponding IMAGE *Xenopus *clones were fully sequenced in both directions with the "PRISM* Ready Reaction Big Dye* Terminator cycle Sequencing Kit" and an automated ABI 310 sequencer (PE Biosystems, Courtaboeuf, France), as recommended by the manufacturer. Multiple amino acid sequence alignments were constructed using ClustalW software [108].

### Phylogenetic analysis

In order to identify orthologous genes, phylogenetic analysis of studied genes was done using the FIGENIX phylogenomic analysis pipeline [109]. FIGENIX allows sequences retrieval, multiple sequence alignments, phylogenetic reconstruction and orthology and paralogy relationships deductions. The phylogenomic task was run using either trout or *Xenopus *amino acid sequence as a query, with the default parameters, and using Ensembl and NCBI-nr databases with search limited to mammals, chicken, *Xenopus *and fish.

### RNA extraction, reverse Transcription and Real-Time PCR analysis

RNA extraction and reverse transcription were performed as previously described for rainbow trout [14]. For *Xenopus*, 5 μg of total RNA were reverse transcribed using 200 units of SuperScript™ reverse transcriptase (Invitrogen, Cergy Pontoise, France) and 250 ng random hexamers (Promega) in a reverse transcription master mix containing 2 mM dNTPs, 50 mM Tris-HCl, 75 mM KCl, 3 mM MgCl_2_, 10 mM dithiothreitol, pH 8.3. Twenty-five units of RNase inhibitor (RNasin, Promega) were added to the reaction. RNA and dNTPs were denatured for 5 min at 65°C, and then chilled on ice before addition of reverse transcription master mix. Reverse transcription was performed at 25°C for 10 min then at 42°C for 50 min followed by a 15-min incubation step at 70°C. For both species, control reactions were run without reverse transcriptase and used as negative controls in the real-time polymerase chain reaction (PCR) study for all target genes.

QPCR was done using a real-time PCR system Step One Plus (Applied Biosystems, Foster City, USA). Reverse transcription products were diluted to 1/25 for trout and 1/20 for *Xenopus*. Triplicates were run for each RT product. Real-time PCR was performed using a real-time PCR kit provided with a SYBR Green fluorophore (Fast SYBR Green Master Mix, Applied Biosystem). Primer concentrations were 240 nM for 18S and 600 nM for all other genes. The hot start enzyme was activated 20 seconds at 95°C, then the amplification was carried out using the following cycle: 95°C for 3 sec; 60°C for 30 sec; 40 times. A pool of reverse transcribed RNA was serially diluted and used to calculate a standard curve. For all studied genes, 18S was used as an internal standard to normalize the signal. After amplification, a fusion curve was obtained according to the following protocol: 10 sec holding followed by a 0.05°C increase, repeated 80 times, and starting at 55°C. Primer sequences of studied genes are presented in supplementary data files (see additional file 6 for trout; see additional file 7 for *Xenopus*). The control reactions were used to calculate background expression level for each gene in order to evidence the tissues exhibiting expression levels significantly above background. Statistical analyses were performed using Statistica 7.0 software (Statsoft, Tulsa, OK). Regarding trout and *Xenopus *data, differences between groups were analyzed using non parametric U Mann-Whitney test and paired samples Wilcoxon test, respectively.

## Authors' contributions

MG performed the microarray data analyses, real-time PCR experimentations, and drafted the manuscript. MG and FC extracted *Xenopus *RNA while JB extracted trout RNA. FC and JB designed and supervised the study, participated in the analyses and candidates selection and in the writing of the manuscript. All authors read and approved the final manuscript.

## Supplementary Material

Additional file 1 **Biological process enrichments of cluster 1 (= genes down-regulated during maturation)**. Gene Ontology functional annotations and biological process term enrichments were performed using the DAVID web-based tool (Database for Annotation, Visualization, and Integrated Discovery) [103-105]. Enrichment analyses were performed using all the annotated genes present on the microarray (8 344 genes) as the reference and using UNIPROT accession IDs as input for the differentially expressed genes. Enriched GO biological process terms associated with each gene list were determined using modified Fisher's exact tests. GO terms with a raw p-value lower than 0.05 are presented. The number of genes associated with each GO term and associated enrichment information (raw p-values, fold enrichment) are indicated for each GO term. Data are sorted by decreasing enrichment fold values. Click here for file

Additional file 2 **Biological process enrichments of cluster 2 (= genes up-regulated during maturation)**. Gene Ontology functional annotations and biological process term enrichments were performed using the DAVID web-based tool (Database for Annotation, Visualization, and Integrated Discovery) [103-105]. Enrichment analyses were performed using all the annotated genes present on the microarray (8 344 genes) as the reference and using UNIPROT accession IDs as input for the differentially expressed genes. Enriched GO biological process terms associated with each gene list were determined using modified Fisher's exact tests. GO terms with a raw p-value lower than 0.05 are presented. The number of genes associated with each GO term and associated enrichment information (raw p-values, fold enrichment) are indicated for each GO term. Data are sorted by decreasing enrichment fold values. Click here for file

Additional file 3 **Phylogeny of *cyp17a1 *in vertebrates including fish**. This npl tree is the fusion of neighbour joining (n), maximum parsimony (p), and maximum likelihood (l) trees calculated with the FIGENIX automated phylogenomic annotation pipeline [109]. The task was run using the *Xenopus *amino-acid sequence as a query. Protein sequence accession numbers for each species are given under brackets on the right of the figure. Evolutionary distances among sequences are represented by the tree structure, where branch length represents evolutionary distance. For each node, bootstrap values are reported for each npl method. Bootstrapping was carried out using 100 replications. Click here for file

Additional file 4 **Phylogeny of *hsd11b3 *in vertebrates including fish**. This npl tree is the fusion of neighbour joining (n), maximum parsimony (p), and maximum likelihood (l) trees calculated with the FIGENIX automated phylogenomic annotation pipeline [109]. Protein sequence accession numbers for each species are given under brackets on the right of the figure. Evolutionary distances among sequences are represented by the tree structure, where branch length represents evolutionary distance. For each node, bootstrap values are reported for each npl method. Bootstrapping was carried out using 100 replications. The task was run using the rainbow trout amino-acid sequence as a query. Click here for file

Additional file 5 **Phylogeny of *apoC1 *in vertebrates including fish**. This npl tree is the fusion of neighbour joining (n), maximum parsimony (p), and maximum likelihood (l) trees calculated with the FIGENIX automated phylogenomic annotation pipeline [109]. Protein sequence accession numbers for each species are given under brackets on the right of the figure. Evolutionary distances among sequences are represented by the tree structure, where branch length represents evolutionary distance. For each node, bootstrap values are reported for each npl method. Bootstrapping was carried out using 100 replications. The task was run using the *Xenopus *amino-acid sequence as a query. Click here for file

Additional file 6 **Primers used for the QPCR study in *Oncorhynchus mykiss***. For each target gene, full name, symbol, GenBank accession number and primers sequences are indicated. Click here for file

Additional file 7 **Primers used for the QPCR study in *Xenopus laevis***. For each target gene, full name, symbol GenBank accession number and primers sequences are indicated. Click here for file
