# Supplementary figures and images for: Comparative transcriptomic analysis of follicle-enclosed oocyte maturational and developmental competence acquisition in two non-mammalian vertebrates
Source: BMC Genomics. 2010 Jan 8;11:18. doi: 10.1186/1471-2164-11-18 (PMC2821372; doi:10.1186/1471-2164-11-18)

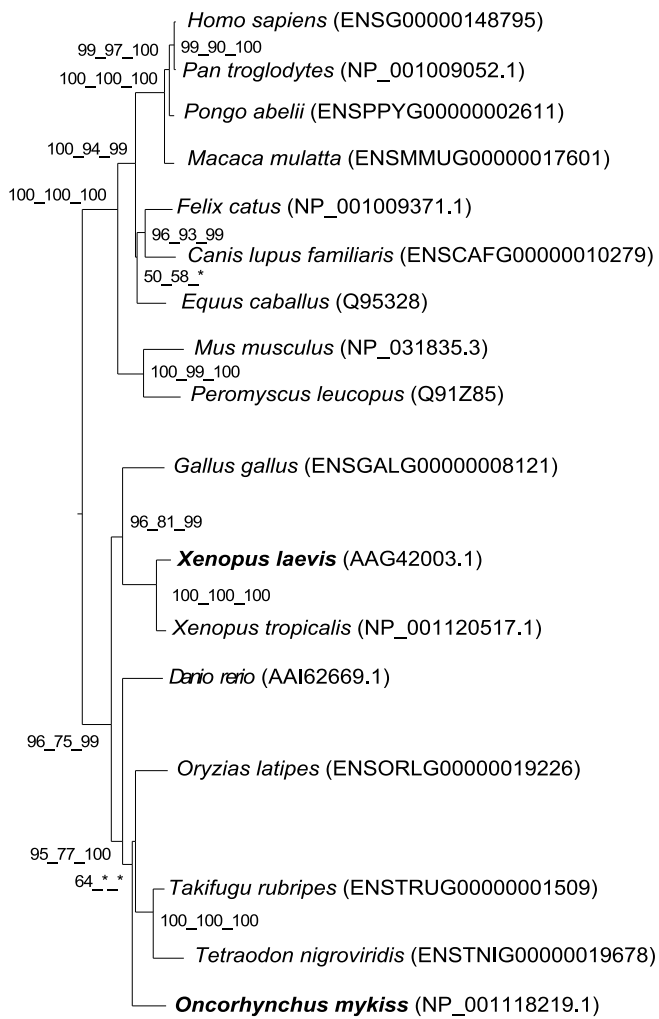

Supplement: Additional file 3 — Phylogeny of cyp17a1 in vertebrates including fish. This npl tree is the fusion of neighbour joining (n), maximum parsimony (p), and maximum likelihood (l) trees calculated with the FIGENIX automated phylogenomic annotation pipeline [109]. The task was run using the Xenopus amino-acid sequence as a query. Protein sequence accession numbers for each species are given under brackets on the right of the figure. Evolutionary distances among sequences are represented by the tree structure, where branch length represents evolutionary distance. For each node, bootstrap values are reported for each npl method. Bootstrapping was carried out using 100 replications. [file 1471-2164-11-18-S3.PDF]

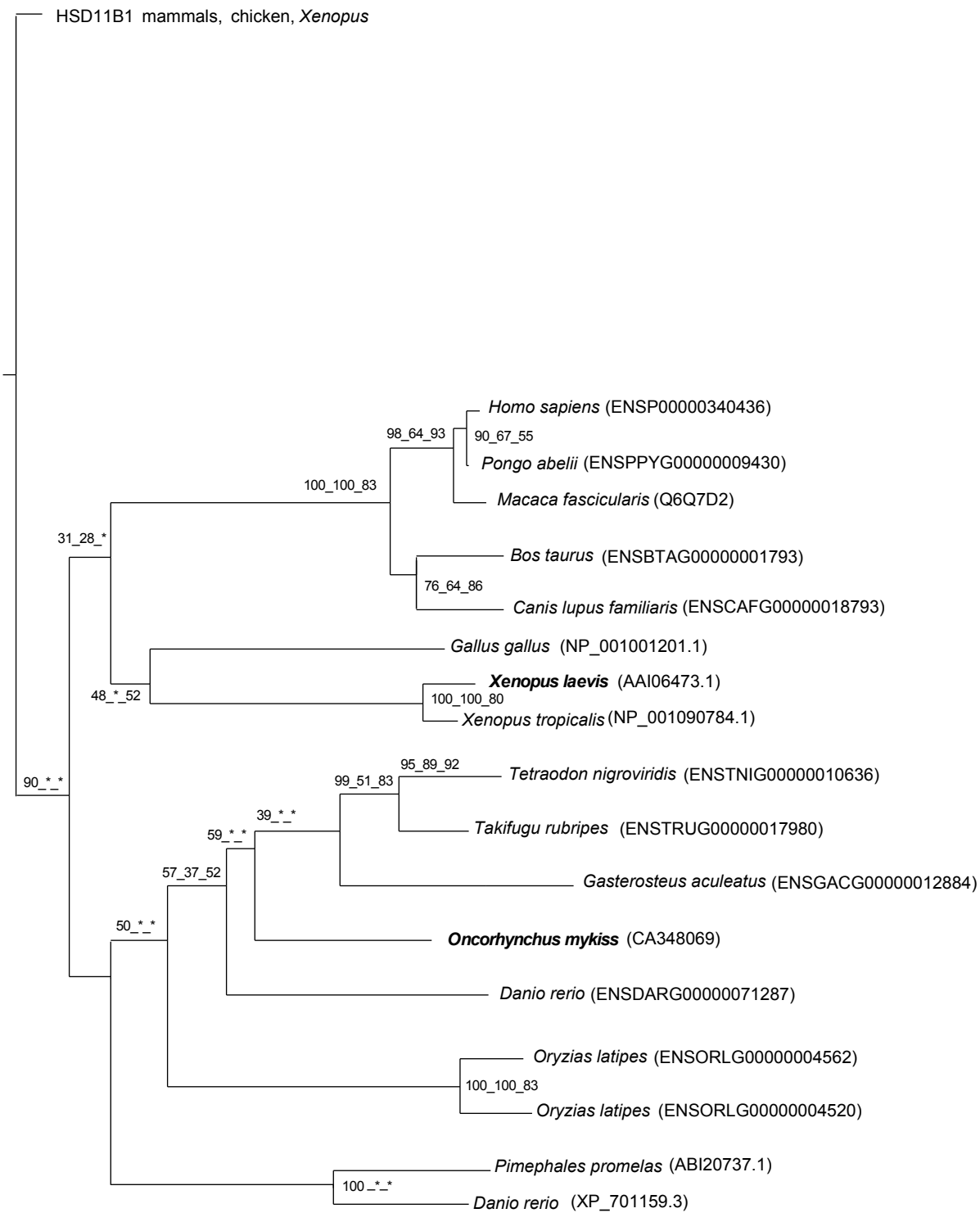

Supplement: Additional file 4 — Phylogeny of hsd11b3 in vertebrates including fish. This npl tree is the fusion of neighbour joining (n), maximum parsimony (p), and maximum likelihood (l) trees calculated with the FIGENIX automated phylogenomic annotation pipeline [109]. Protein sequence accession numbers for each species are given under brackets on the right of the figure. Evolutionary distances among sequences are represented by the tree structure, where branch length represents evolutionary distance. For each node, bootstrap values are reported for each npl method. Bootstrapping was carried out using 100 replications. The task was run using the rainbow trout amino-acid sequence as a query. [file 1471-2164-11-18-S4.PDF]

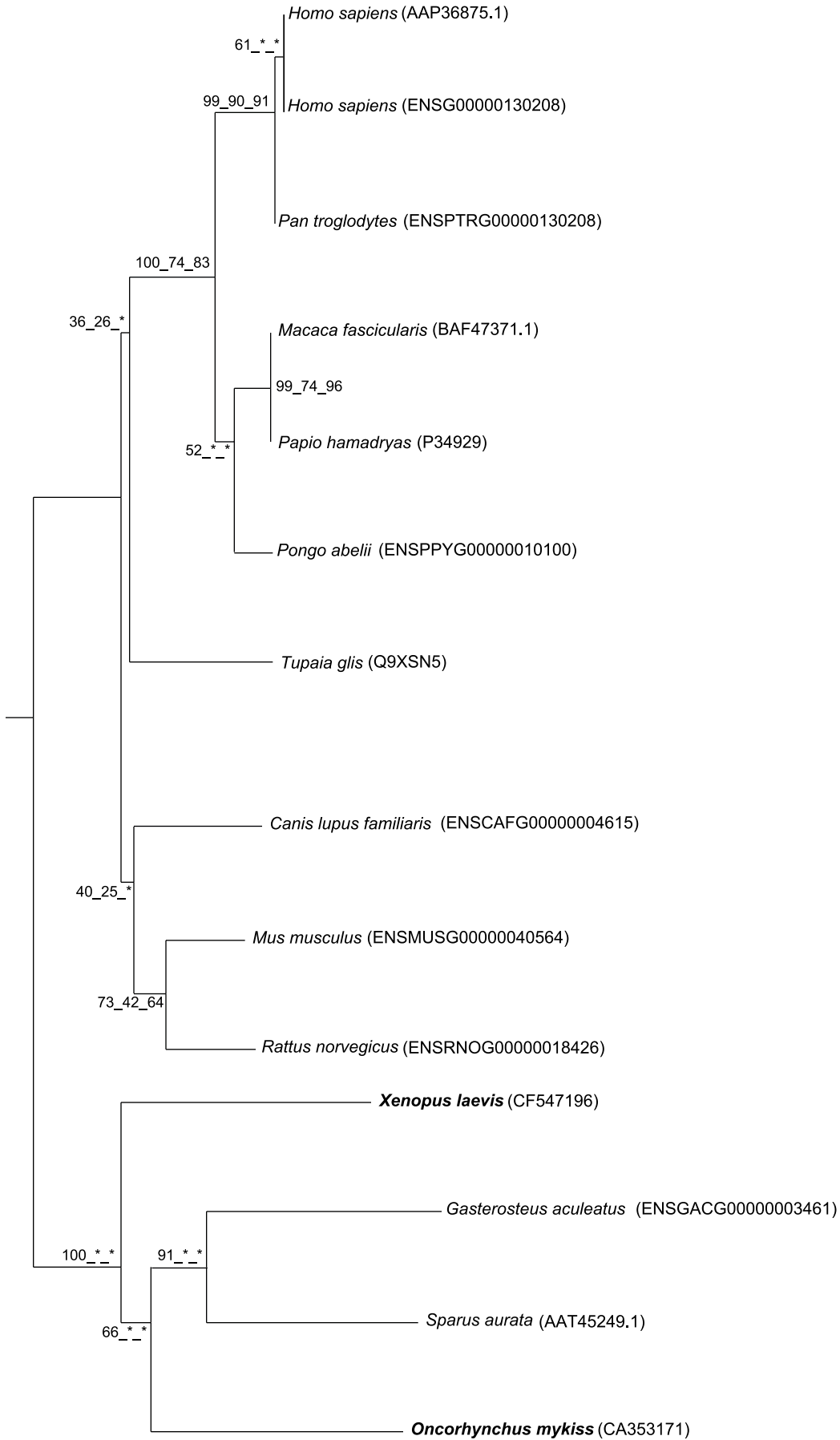

Supplement: Additional file 5 — Phylogeny of apoC1 in vertebrates including fish. This npl tree is the fusion of neighbour joining (n), maximum parsimony (p), and maximum likelihood (l) trees calculated with the FIGENIX automated phylogenomic annotation pipeline [109]. Protein sequence accession numbers for each species are given under brackets on the right of the figure. Evolutionary distances among sequences are represented by the tree structure, where branch length represents evolutionary distance. For each node, bootstrap values are reported for each npl method. Bootstrapping was carried out using 100 replications. The task was run using the Xenopus amino-acid sequence as a query. [file 1471-2164-11-18-S5.PDF]
