# Supplementary material for: Comparative transcriptomic analysis of follicle-enclosed oocyte maturational and developmental competence acquisition in two non-mammalian vertebrates
Source: BMC Genomics. 2010 Jan 8;11:18. doi: 10.1186/1471-2164-11-18 (PMC2821372; doi:10.1186/1471-2164-11-18)
Supplement: Additional file 6 — Primers used for the QPCR study in Oncorhynchus mykiss. For each target gene, full name, symbol, GenBank accession number and primers sequences are indicated. [file 1471-2164-11-18-S6.PDF]

target genes	symbol	GenBank#	Forward sequence	Reverse sequence
Aromatase	cyp19a1a	BX083177	CTCTCCTCTCATACCTCAGGTT	AGAGGAACTGCTGAGTATGAAT
Cytochrome P450 17A1	cyp17a1	BX072662	ACAGTAACCACAGACCTGTTG	CCTACAGATAATCTTCTCGAT
RNA-binding region-containing protein 39	rbm39a	BX081888	GGGCCATTGCTATTGGTG	GCTGGGATGGCAAGTTCATA
A disintegrin and metalloproteinase domain 8	adam8a	BX871415	GGACTGATGTGCTGCAAGAA	GGAACATTGGGTTCAACTGG
A disintegrin and metalloproteinase domain 22	adam22	CA363158	CCCGACTAGGAGAGTTGCAG	ATCATCACATGACCCCCACT
Protein kinase C delta type	prkcd	CA372310	GGAGGAGACCTGATGTTCCA	GATCCCTTTGGAATGCAGAA
Serum/glucocorticoid related kinase 2	sgk2	CA387850	GACTATGTCAACGGGGGAGA	ACTGGCTACCTCAGCAGCAT
Serine protease 23	prss23	BX087643	ACTGCCGAGAAGGATGAAGA	CCTCAGCAAGGGAAGTGAAG
Regulator of G-protein signaling 18	rgs18	BX876662	AAGGCACCGAGAATGAAAGA	TTTTCATGGGAGCCTGACTT
Forkhead box O5	foxo5	BX885992	AGTTGGACGTGGCCATAGAC	TCAGTGTCGTTGCTTTCTGG
Cytidine monophosphate-N-acetylneuraminic acid hydroxylase	cmah	BX878414	GGAGGCCTGTTCATCAAAGA	CCTGTGTGAAGCTGTCAGGA
Steroidogenic acute regulatory protein	star	BX079021	ATGCCCTTTGACCTTTCGAT	GCCTTTGATGGAGAAAGTGG
Growth arrest and DNA-damage-inducible protein beta	gadd45bii	CA363171	GTTACAGGACGTGGGCAACT	TCTCAGCGAGGGTCGTTAGT
Apolipoprotein CI	apoc1	CA353171	GCTGTCCCCAATCTTTTCAA	GGCTGAGAAGACCATTGAGG
11-beta-hydroxysteroid dehydrogenase-like	hsd11b3	CA348069	ACATGGCCAATCCTTCTGAC	AATGGGCTGGAACCTATGTG
Receptor-type tyrosine-protein phosphatase F	ptprf	CA360891	CGTGAGATTCGTCAGTTCCA	GTAGGAAGGCCAGGATAGGG
Dystrophin	dmd	CA377239	TCTGTCCATCCAGAGCAGTG	TCATCTCCAGGTCACCCTTC
Cyclin L1	ccnl1	BX863114	TGCTGGACTTGGAGACAACA	ACGCATTCATTCCTTCTTCC
Transferrin receptor protein 1	tfr1	BX860777	GCACCTGGATGTGGAGAAAT	GGACAGACAGCGACTTAGGC
C-ETS-2 protein	ets2	CA368141	CTGGCCTCAACTGTGTCTCA	CCTTGGAGAAGCCACTGAAG
ETS translocation variant 5	etv5	BX870637	CAGCGACAGTGCCTATGAGA	TCCTTGGGGTGTTGGTAGAG
Gap junction Cx32.2 protein (Connexin-32.2)	Cx32.2	BX082081	GGTTGACAATGGCATGTTGAAG	CAACTGGGTCATATACGTGCGT
Tight junction protein ZO-1	tjp1	BX872029	ATAGCTCAGCGATGGAGGAA	TCTGGAAGTGAGGGTTGTCC
Claudin-11	cldn11	BX301535	TTTCAACCGCCCTCTATCAC	CCCCAGGAGGATCCAGTTAT
Tescalcin	tesc	BX877446	TGCACATCACAGAGGAGGAG	GCCGTCACTGTCTGTGTCAT
7-dehydrocholesterol reductase	dhcr7	BX884545	CCTCTCCTATGCAGCCAAAC	ACCATGCCTCATTCCAGAAG
