# Supplementary material for: Comparative transcriptomic analysis of follicle-enclosed oocyte maturational and developmental competence acquisition in two non-mammalian vertebrates
Source: BMC Genomics. 2010 Jan 8;11:18. doi: 10.1186/1471-2164-11-18 (PMC2821372; doi:10.1186/1471-2164-11-18)
Supplement: Additional file 7 — Primers used for the QPCR study in Xenopus laevis. For each target gene, full name, symbol GenBank accession number and primers sequences are indicated. [file 1471-2164-11-18-S7.PDF]

target genes	symbol	GenBank#	Forward sequence	Reverse sequence
Aromatase	p450arom-A	NM_001085653	CTTCGGGGAGCAGTTTGTTA	CCTGTCATGCACAACCATCT
Cytochrome P450 17A1	cyp17a1	AF325435	TGCACAAATTAGAAAGGCAAAA	AGCACAACGTGTGTTGGAAA
RNA-binding region-containing protein 39	rbm39	BC077813.1	GAGAGAGTGTCATCGGTGCAT	AGGCAGCATAGGCAATGAAT
A disintegrin and metalloproteinase domain 13	adam13	NM_001087445	TCCCAATGGCTGTATACTGGA	TTAAGGAAATAATGCCTTCAGCA
A disintegrin and metalloproteinase domain 11	mdc11b	NM_001087444.1	GACCCTCTTTACCGCTGTTG	TCTGCTGTGTCCTTGTAGCC
Protein kinase C delta type	pkc-delta2	NM_001090993	TAACGCGTCACTCCTTGTGT	CAGATGTGAGGGGCTTCATT
Serum/glucocorticoid related kinase 1a	sgk1a	NM_001090340	AAATAGCCAGTGCGCTAGGA	GTGCCATTGGGCTCTATGTT
Serine protease 23	prss23	CD255705	CTATTCCAACGGAAGCCGTA	GCCGTATCTCTGCCTCTTTG
Regulator of G-protein signaling 18	rgs18	NM_001092321	ACGGCACAGAGTTGATGATG	TGCGATTGCATAAAACAAGG
Forkhead box O3	foxo3	NM_001092949	CAAATCTCCAGTGCAAATCG	TGCTCTATGGAAGACCCATGT
Cytidine monophosphate-N-acetylneuraminic acid hydroxylase	cmah	NM_001086828	ACCCACCAGACAGTTTTTGC	ATCCCAAGGATTTGGAGGAC
Steroidogenic acute regulatory protein	star-A	AF220437	ACGGAGAACTGGTGGACAAC	CTCTGCTGCTTTCTCGTGTG
Growth arrest and DNA-damage-inducible protein beta	gadd45b	DT070441	TTAAGGGCACCCACTGACTC	CCTCCACTGCATTCTGGTTT
Apolipoprotein CI	apoc1	DQ096911	CACCTAGACGTGACCAGCAA	GCTGACATGACTGGAACAA
11-beta-hydroxysteroid dehydrogenase-like	hsd11b3	BC106472	ATTCAAAAGGCAGTGTGGTT	TTGGTTGCTGACAGATGAAGA
Receptor-type tyrosine-protein phosphatase F	xptp-d	NM_001090381	AAAAAGACATTTTCTGGGTGGA	ACCATCCCAGTGAGAGCAAT
Dystrophin	dmd	X99700	ATGACTTGGGAAGAGCGATG	GTCGTGATTGGAACGGTCAT
Cyclin L1	ccnl1	BC073707	TCAATTTACACAACACGGACA	TGGGAGAAGCCTTTTAATACACA
Transferrin receptor protein 1	tfr1	CA790915	TCCATCTCAAGGCATTTTCC	TCAGCGTTTGCAGTATCACC
C-ETS-2 protein b	ets2b	X52635	GATGGATGGCGCTTATTGTT	CCTTTAGGTGGGACGCAATA
XER81	xer81	AF057565	ATCCAGCAGCAGGAAAAGAA	AGCTTCTGCAAGCCATGTTT
Gap junction Cx32.2 protein (Connexin-32.2)	cx32.2	DQ096928	GGGGCAGGAGAAAGGAGAT	CTGCCCTCTCCGTCATGT
Tight junction protein ZO-1	tjp1	BC088825	TGCAACTTCCAGTAGTTCGTTC	CACTTCTCTGGTCAGGCACA
Claudin-11	cldn11	CB591807.1	TACGTGCAGAAAGCTGGATG	ATGTGAGCAGGCAGTGTCAG
Tescalcin	tesc	NM_001095029	ATGGAAAAGCGGTTAGTTGC	CCTTTTCTGCTGCCCATTAC
7-dehydrocholesterol reductase	dhcr7	NM_001087087	CACTAACGGCAACTGCAAGA	CCCAGAACCCTGAAATCATC
